# Three-dimensional reach trajectories as a probe of real-time decision-making between multiple competing targets

**DOI:** 10.3389/fnins.2014.00215

**Published:** 2014-07-23

**Authors:** Jason P. Gallivan, Craig S. Chapman

**Affiliations:** ^1^Department of Psychology, Centre for Neuroscience Studies, Queen's UniversityKingston, ON, Canada; ^2^Faculty of Physical Education and Recreation, University of AlbertaEdmonton, AB, Canada

**Keywords:** reaching, decision-making, motor, cognition, vision

## Abstract

Though several features of cognitive processing can be inferred from the discrete measurement [e.g., reaction time (RT), accuracy, etc.] of participants' conscious reports (e.g., verbal or key-press responses), it is becoming increasingly clear that a much richer understanding of these features can be captured from continuous measures of rapid, largely non-conscious behaviors like hand or eye movements. Here, using new experimental data, we describe in detail both the approach and analyses implemented in some of our previous studies that have used rapid reaching movements under cases of target uncertainty in order to probe the features, constraints and dynamics of stimulus-related processing in the brain. This work, as well as that of others, shows that when individuals are simultaneously presented with multiple potential targets—only one of which will be cued after reach onset—they produce initial reach trajectories that are spatially biased in accordance with the probabilistic distribution of targets. Such “spatial averaging” effects are consistent with observations from neurophysiological studies showing that neuronal populations in sensorimotor brain structures represent multiple target choices in parallel and they compete for selection. These effects also confirm and help extend computational models aimed at understanding the underlying mechanisms that support action-target selection. We suggest that the use of this simple, yet powerful behavioral paradigm for providing a “real-time” visualization of ongoing cognitive processes occurring at the neural level offers great promise for studying processes related to a wide range of psychological phenomena, such as decision-making and the representation of objects.

## Introduction

Object-directed behavior requires that the action-relevant features of an object, such as its location or orientation, be transformed into coordinated motor commands specifying the appropriate set of movements (Jeannerod et al., 1995; Jeannerod, 1997). Traditional serial models of action planning have posited that the final target of an action must be selected prior to the associated motor commands being specified (i.e., action selection before specification, see Miller et al., 1960; Sternberg, 1969; McClelland, 1979). Recent neural work examining motor planning in cases of target uncertainty, however, has shed considerable light on the mechanisms involved in selecting between targets and mounted significant challenge to traditional serial views of action planning. For instance, in the context of reach planning, neural recordings from dorsal premotor cortex (PMd), a structure involved in sensorimotor transformations for reaching, show that neurons in the region appear to simultaneously represent multiple potential reach targets (i.e., leftward and rightward targets) in parallel prior to one of those targets being selected as the final action target (Cisek and Kalaska, 2002, 2005). Likewise, in the context of grasp planning, neural recordings from the anterior intraparietal area (AIP), a structure involved in sensorimotor transformations for grasping, show that neurons in the region appear to simultaneously represent in parallel multiple potential grip types (i.e., possible precision- and power-grip movements) prior to the final grip type being selected (Baumann et al., 2009). Furthermore, in the context of eye movement planning, neural recordings from the superior colliculus (SC) and lateral intraparietal area (LIP), subcortical and cortical structures involved in the planning and generation of saccades, respectively, show that neurons in these regions also appear to simultaneously represent in parallel multiple potential saccade locations (i.e., leftward and rightward targets) prior to the final saccade location being selected (Basso and Wurtz, 1997; Platt and Glimcher, 1997). Taken together, this accumulating neural evidence suggests that, in cases of target uncertainty, brain structures involved in planning actions with a particular movement effector (arm, hand, or eyes) automatically represent multiple competing actions available to that effector before the decision is made between those competing actions. These neural findings raise the important question: Is there evidence for this “internal” competition between potential actions in behavior, and, if so, under what types of task conditions might it be observed?

It has become increasingly clear that the specification of multiple competing actions and the ongoing dynamics of deciding between those actions can be captured from continuous measures of rapid reach and eye movements. It was initially the seminal work of Ghez et al. (1997) who showed that individuals, when presented with two competing reach targets, often launched their hand toward the midpoint, or spatially averaged location between those targets. In these experiments, participants were presented with more than one potential reach target and were required to initiate a reach response at latencies near the time when one of those targets would be selected as the final target. The “spatial averaging” behavior seen in this work has also been frequently observed in eye movements. At early saccade latencies, when individuals are simultaneously presented with a saccade target and a similar distractor non-target, the eyes will often veer toward the distractor, or follow a path that averages between the distractor and target locations (Findlay and Walker, 1999; Arai et al., 2004; McSorley et al., 2006; Van Der Stigchel et al., 2006; Walker et al., 2006; McSorley and McCloy, 2009).

Capitalizing on this general phenomenon, recent work in psychology and sensorimotor neuroscience has begun documenting the effectiveness of continuous movement trajectories in revealing rich and highly detailed cognitive processing information. For instance, Song, Nakayama, and colleagues (Song and Nakayama, 2008; Song et al., 2008, see Song and Nakayama, 2009 for review) have shown using a color-oddity task the powerful attraction that competing non-target stimuli have during reaching movements. In these tasks, individuals perform reaches toward an odd-colored target in the presence of distractors (e.g., a single red target amongst several green distractor targets). On trials when the colors of targets and distractors are randomly switched (e.g., now a single green target among red distractors) there is heightened competition between the stimuli, and reach trajectories will often be initially biased in the direction of distractors before correcting to the correct (odd-colored) target location. Similarly, Spivey et al. (2005) showed that when participants are instructed to use a computer mouse to click on a certain picture (e.g., picture of a car), initial mouse-cursor trajectories are more deviated toward the midpoint of two pictures that are phonologically similar (e.g., a picture of a “car” on the left and a “cat” on the right) than those that do not share phonological features (e.g., pictures of a “car” and a “house”). In the field of social psychology, this mouse-cursor paradigm and spatial averaging phenomena has been exploited to examine individuals' internal representation of “true” or “false” statements (Duran et al., 2010, 2013; Dale and Duran, 2011), their certainty of a statement (McKinstry et al., 2008), and their categorization of race and gender (Freeman et al., 2008, 2010; Freeman and Ambady, 2009; see Freeman et al., 2011 for review), to name but a few examples. Finally, in work closely related to that described here, researchers have used rapid reach trajectories and/or the endpoints of reaches in order to examine how target uncertainty (e.g., Kording and Wolpert, 2004; Hudson et al., 2007) or value (see Trommershauser et al., 2008 for review) affects human movements. Regardless of the specific application of the paradigm, however, the basic observation is always the same: Stimuli and/or choices that elicit competing representations tend to generate greater spatial averaging effects and, notably, such effects do not actually depend on which target is ultimately selected by the participant on that particular trial. Thus, across several different fields of research and applications of the general paradigm, continuous trajectory measures clearly provide the experimenter with a unique window on ongoing internal cognitive processes and have the capacity to reveal important insights into how a wide variety of stimuli are represented in the brain.

**Figure 1:**
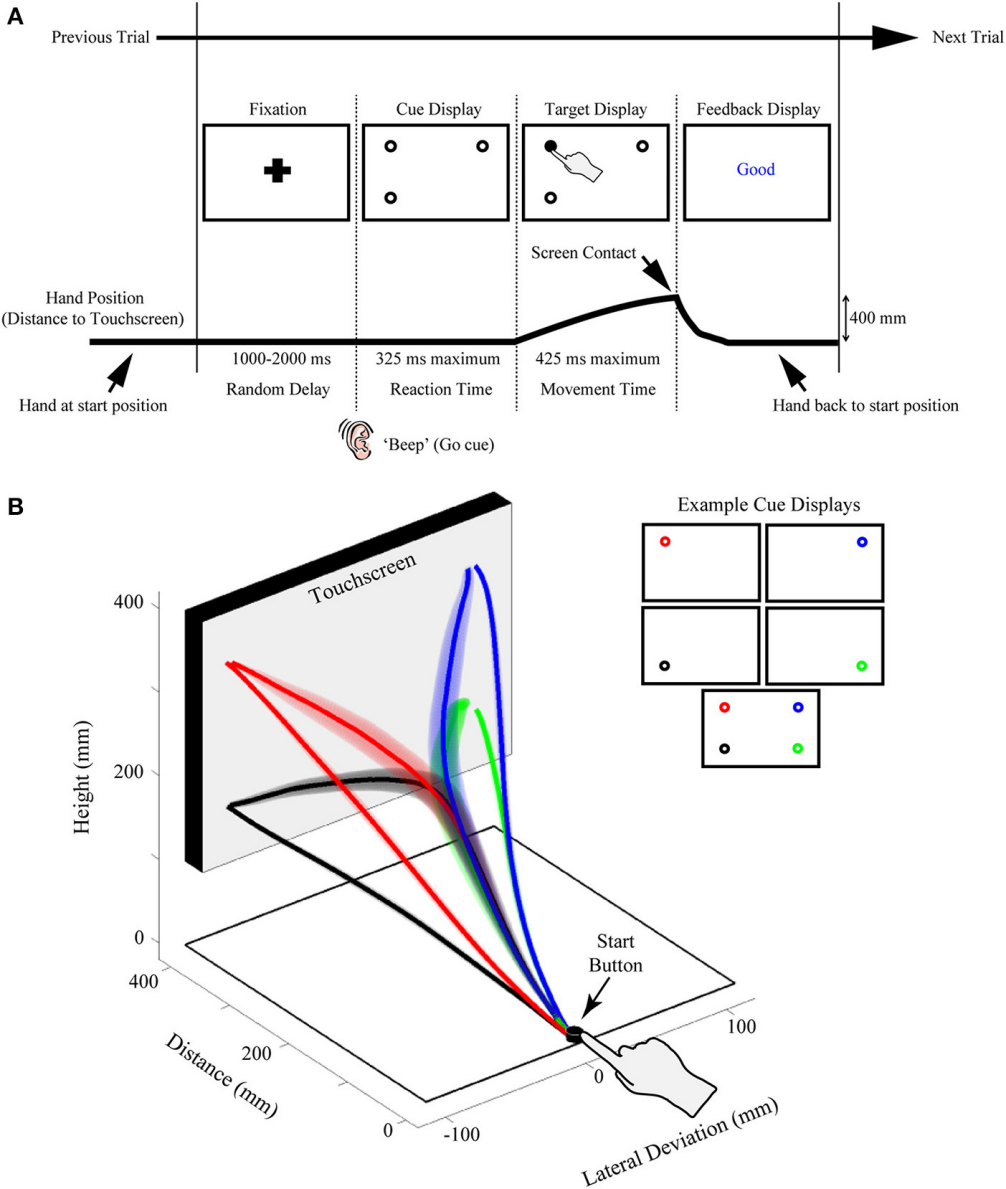
**Illustration of the rapid reach task and examples of subjects' average arm trajectories. (A)** Sequence of events for a representative single trial. Following the presentation of a fixation cross for a variable delay period, potential targets (unfilled circles) were displayed in one to four possible locations on the touchscreen (the bottom right potential target location was not presented on this particular trial). In the task, subjects were required to release a start button at the onset of a “beep” go cue (which was simultaneous with the onset of the cue display) and begin reaching toward the target display within 325 ms. Immediately following button release, one of the targets in the display was selected (filled-in black) and subjects were required to correct their reach trajectory and touch the screen at that location within 425 ms. Importantly, all potential targets in the cue display had an equal probability of being selected as the final target. Subjects then received text feedback on the screen following the completion of a trial (Feedback display). The thick black line below the display sequence denotes the linkage between the subject's movement and order of events in the trial. **(B)** Three-dimensional view of the experimental setup with group-averaged reach trajectories (*n* = 24) for the example cue displays shown at right. Reach trajectories are color-coded according to the final target's position (and thus, reach endpoint). Trajectories that initially aim toward the midpoint of the touchscreen are trials in which all four potential targets were presented simultaneously (example cue display at bottom right). Shaded areas around the darker trajectories represent average standard errors across subjects. Note for this plot, and all subsequent trajectory plots, the x-axis is shown at twice scale.

Recently, we and others have begun exploiting the sensitivity of continuous reach data in general, and subtle shifts in the heading of the initial component of the reach trajectory in particular, to provide a behavioral index of both the temporal dynamics and constraints of target feature processing. One key feature of our reaching paradigm is its use of target uncertainty. This can be achieved by requiring participants to initiate a movement before one of several possible targets is cued (see Figure 1A). As mentioned above, the effects of target uncertainty on reach behavior have been well explored in previous work (e.g., Ghez et al., 1997; Hudson et al., 2007). Hudson et al. (2007) presented multiple potential reach targets (up to nine vertical bars) made up of white and gray pixels. Notably, the density of white pixels denoted the probability of that target being selected as the final target, thereby allowing the authors to precisely vary target uncertainty. Similar to many of the effects reported above, the authors found that the hand moved along a path that optimally averaged between the different possible outcomes. In our typical task, we manipulate target uncertainty not through some secondary target feature like pixel density (which requires that participants learn the association between density and outcome) but rather through changing the number and location of multiple equiprobable potential targets in a display. An advantage of the latter approach is that, first, the task is very intuitive and easy for participants to comprehend (i.e., each target has an equal likelihood of being selected) and, second, it also provides the flexibility of using secondary target features (e.g., target salience, probability, learned associations) to explore other dimensions of stimulus competition, and, in some cases, to directly pit target quantity vs. those secondary features (e.g., Wood et al., 2011; see sections Extensions of Procedure and Extensions of the Task and Future Work for discussion of examples).

Another key feature of our paradigm is the rapid nature of the reach responses required: Participants are typically required to initiate a reach movement toward the target display within 325 ms of its presentation [reaction time (RT) criteria] and then correct their reach trajectory to the cued target location within 425 ms (movement time (MT) criteria; see Figure 1A). Together, this stringent timing criteria and the fact that the final target location is only cued following movement onset, requires that participants—in order to be effective at the task—must prepare an initial movement trajectory that takes into consideration all potential target options.

In our original work we found that when individuals are presented with multiple potential targets and are required to rapidly act upon those targets without knowledge of which target will be the final target for action, they incrementally shift their initial reach trajectory vectors in accordance with the probability of acting at each target location (Chapman et al., 2010a); that is, initial reach trajectories are biased in accordance with the midpoint of the target distribution. Further work using this same paradigm showed that these initial rapid trajectory effects, in addition to being influenced by the spatial position of potential targets, are also influenced by previous trial history (Chapman et al., 2010b) as well as the visual salience of the competing targets in the display (Wood et al., 2011). We have gone on to further show that there also appears to be a capacity limit of 3–4 competing targets that can be encoded during reach planning (Gallivan et al., 2011), a capacity limit often seen in visual short-term memory and object tracking tasks. With specific relevance to the field of numerical cognition, we have also shown using the rapid reach task that individuals appear to use symbolic and non-symbolic quantity information in fundamentally different ways to form action plans based on that quantity information (Chapman et al., 2014). Finally, some of our most recent work using the rapid reach paradigm has provided compelling evidence that visual scene parsing for perception depends on separate mechanisms than that which supports the planning of rapid and efficient target-directed movements (Milne et al., 2013). Taken together, this body of work suggests that rapid initial movement trajectories, under cases of target uncertainty, reflect the simultaneous preparation of multiple reaches to potential targets. This is consistent with neurophysiological studies in sensorimotor brain areas showing the simultaneous representation of multiple potential actions before deciding between them (see Cisek and Kalaska, 2010 for review) and it may provide a mechanism through which the visual-motor system is able to make rapid adjustments and online corrections when the initial target changes (Brenner and Smeets, 1997; Gomi, 2008; Resulaj et al., 2009; Nashed et al., 2014). At a more general level, this work suggests that researchers can use movement trajectories as a tool to sensitively measure the bias generated toward (or away from) competing stimuli in almost any context. As discussed, this can extend from biases generated from the low-level physical parameters of a stimulus, like its salience (e.g., Wood et al., 2011), all the way up to those elicited by higher-level cognitive concepts, like the subjective truth value of a particular statement (e.g., Duran et al., 2010). From this vantage point, the reach paradigm, and motor behavior more generally, can be easily employed to study cognitive processes across a diverse range of research domains, spanning from early visual processing to complex decision-making (for reviews, see Cisek, 2012; Wolpert and Landy, 2012) and from one's social categorizations to their individual preferences (for review, see Freeman et al., 2011).

In the current paper we provide a detailed overview of our rapid reach paradigm and highlight its implementation through a new experiment. Though this experiment is designed to explore biases in the three-dimensional hand paths of participants based on the spread of potential targets is both the horizontal and vertical dimension (biases not explored particularly rigorously in our previous work), the overarching goal of this paper is to provide researchers with sufficient detail and working knowledge so as to enable the implementation of the paradigm (and its variants) in their own work. To this end, we use the experimental data only as an opportunity describe, in detail, the sophisticated functional data analysis (FDA) methods and more conventional non-functional statistical techniques that we have previously found to be critical in measuring and summarizing subtle trajectory deviation effects. In our discussion, we also describe some of the merits and potential modifications of the rapid reach paradigm that can be readily exploited by investigators to explore a wide range of sensorimotor and psychological phenomena.

In line with these stated goals, the following Methods and Results sections have been split into two subsequent categories: (1) General Experiment Information about the rapid reach paradigm and, (2) Current Experiment Information, in which we describe the specific implementation and data analyses for the new experiment being presented.

## Materials and methods

### General participant information

For a typical rapid reach experiment, we have found that analyzing data from between 20 and 30 participants produces sufficient statistical power to infer differences in reach trajectories across conditions. Given that our analysis and subsequent interpretation of our reach findings rests on participant's meeting strict temporal and spatial demands, we have found that approximately one in four participants' data are not suitable for analysis. To account for this, we typically collect data from between 30 and 40 participants. As explained below, we apply rigorous trial screening to ensure that only those trials in which there is both a rapid initiation of the reach and an accurate final reach endpoint are included in our analysis. Once trials failing to meet these requirements have been removed, any participant with less than 50% of their original data remaining is removed from analysis. In addition, to ensure that for any given experimental condition we have a reliable estimate of participant performance, we routinely enforce that each participant must have at least 4–8 good trials for each experimental condition (the specific number of “good” trials is determined for each experiment separately since experiments can differ in the number of repetitions per condition). Any participant who fails to meet this performance criterion is also removed from analysis. Given the general difficulty of the task and the aforementioned performance requirements across each condition, during testing we have found it necessary to repeat each experimental condition at least 10 times.

In our previous work employing this paradigm, we have tested predominantly undergraduate student populations (average age ~21 years) with normal or corrected to normal vision. In addition we have only tested individuals who self-identify as right-handed or are right-handed as measured by the Edinburgh handedness questionnaire (Oldfield, 1971). In turn, we have only measured movements of the dominant right hand in this population. These choices are purely pragmatic; it is much easier to find right-handed participants in an undergraduate population and their performance is better when using their dominant hand. We discuss in the Results section below (see section Right Hand Bias) some of the implications that testing this population might have on interpreting rapid reach trajectories.

### Current study participant information

In the particular study presented in the current paper, we collected data from 30 right-handed participants (16 females, average age: 21.1) with normal or corrected to normal vision from the University of Western Ontario undergraduate student population. We removed six participants from the analysis for failing to meet performance criteria (at least 50% of total trials and at least six trials per condition, as described above), leaving 24 participants whose data was included.

### General procedure information

We record rapid reach movements using an infrared marker (or two) placed on the right index finger of our participants. In our previous work, we have used different motion trackers (e.g., Optotrak, Optitrak) and a range of sampling frequencies from 60–200 Hz. As depicted in Figure 1B, participants usually reach from a start button to a large (30+ inch) touch screen (60 hz frame rate) located at 40 cm distance. We use custom Matlab scripts using Psychtoolbox (Brainard, 1997; Pelli, 1997; Kleiner et al., 2007) to control the display. Example trial timing and display configurations can be seen in Figure 1A. Trials begin with participants holding down the start button and fixating a cross centered on screen. After a variable delay of 1000–2000 ms, an auditory “beep” signals when the fixation is replaced by a cue display. Cue displays can consist of almost any visual stimulus imaginable (see section Extensions of Procedure), but we have most commonly used displays consisting of multiple potential targets depicted as 2 cm diameter black outlined circles (4 pixel width) on a white background. We have used a variety of spacing for our potential targets, but we usually place targets such that one cluster of targets has its mean ~10 cm to the left of fixation and another has its mean ~10 cm to the right of fixation. We have also manipulated the height of target locations, as is explored in the specific experiment described here.

The onset of the cue display (coincident with the beep) also acts as a go-signal, instructing participants to initiate a reach movement toward the cue display as rapidly as possible. To enforce rigid RT criteria, participants are required to release the start button after 100 ms has elapsed (to prevent anticipatory responses) and before 325 ms. Upon movement onset (i.e., button release) one of the potential targets in the cue display is selected as the final target (by filling-in black), creating the target display. Unless otherwise manipulated, all potential targets in the cue display have an equal probability of filling-in. Thus, and most critical to this paradigm, participants are required to rapidly plan and initiate their movements under the assumption that all targets in a cue display could be the final target of action. This creates an ideal situation for competing motor plans to be expressed early in the reach movement. To enforce rapid and accurate movement execution (e.g., to avoid potential two-step reaches or poorly aimed reaches) participants are further required, following button release, to reach and touch the cued target within 425 ms, and to have an endpoint within a 6 cm square centered on the cued target.

Participant task performance is reinforced via text feedback displayed on the screen after every trial. There are four possible types of errors that cause the following text to be displayed at the center of the screen: *Too Early* (if the start button is released within 100 ms of the beep go-signal), *Time Out* (if the start button is released more than 325 ms after the beep go-signal), *Too Slow* (if the screen is not touched within 425 ms of movement onset), or *Miss* (if subjects do not touch within a 6 × 6 cm box centered on the cued target position). *Good* is displayed on all trials without errors. On *Too Early* and *Time Out* trials, the final target is not displayed and the trial is immediately aborted. These trials are not analyzed.

As mentioned in the Participant section, we typically repeat a given condition 10 or more times, and usually include 20–40 unique conditions in an experiment. In most cases a single experimental condition denotes a specific cue and target display relationship (e.g., a single experimental condition, in the study included here, would be a four-target trial in which the bottom left target was cued at reach onset). This typically results in experiments comprising 400–600 trials, which are usually separated into ~10 blocks of 40–60 trials each (the separation of trial blocks helps prevent fatigue by allowing participants to have breaks in between). To familiarize participants with the task and timing, prior to beginning the experiment, we typically provide one or two full practice blocks (this practice data is not analyzed).

### Current study procedure information

In the current experiment targets were placed in four positions on the touch screen: 9 cm to the left and right of fixation and 9 cm higher or lower than fixation, arranged like corners of a square (see Figure 1B for an example). All possible cue displays containing one, two, three, and four targets were presented except for two target configurations along the diagonal (i.e., top-left/bottom-right cue display and top-right/bottom-left cue display). This resulted in 13 unique cue displays. For each cue display every potential target was selected as the final target equally often. This resulted in a total of 28 unique experimental conditions (cue display + target display). Each condition was repeated 20 times for a total of 560 experimental trials, administered in 10 blocks of 56 trials each. Participants completed one practice block (56 trials), which was not included in the analysis.

For the current experiment, we used an Optotrak motion tracking system (Northern Digital Instruments; Waterloo, Ontario, Canada) to collect trajectory data (at 150 Hz) from two infrared markers attached to the right index finger of participants. The task and timing used in the current experiment are identical to those explained in the General Procedure section and are depicted in Figure 1A.

### Extensions of procedure

**Figure 2:**
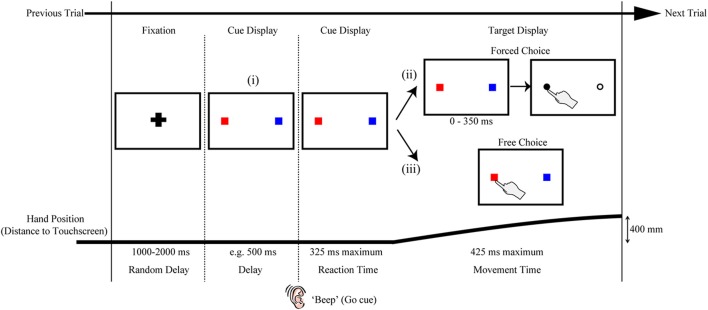
**Variations on the rapid reach task**. The sequences of events are structured similarly to that shown in Figure 1A. Note that the red and blue squares in the target displays are only used here as placeholders; in principle, a wide variety of stimuli (e.g., numerals, shapes, pictures, etc.) can be positioned at these placeholder locations and the resulting reach trajectories toward these stimuli can be used as an index of the level of competition between those stimuli. The variations on the task noted in (i, ii, or iii) are meant to be mutually exclusive for a given trial. (i) One variation on the task can be to include a delay interval between the Fixation and Reaction Time epochs of the trial. In effect, the inclusion of the delay interval will allow participants additional time (in the example here, 500 ms) to view the stimuli before being required to immediately act toward those stimuli. This can be beneficial, for example, when the processing of the stimuli requires additional cognitive resources and/or remembered associations, or when determining the timeline over which a particular bias is generated. (ii) Another variation on the task can be to postpone target selection until much later into the movement (this is denoted by the additional 0–350 ms delay before target selection). In effect, this additional delay would even further limit the amount of target display processing time available to the subject (and correspondingly require much later trajectory corrections to the final target). This can be beneficial, for example, when an experimenter wishes to examine the precise timing at which target display information can be used to influence reach trajectories online. (iii) Still another variation on the task can be to let the participant themselves simply choose which target they would like act on (Free Choice; note that this differs from the “Forced Choice” or selected target paradigms shown in both (ii) and Figure 1A). The resulting rapid free choice will allow researchers to collect conventional measures of choice preference (i.e., the option selected) and the corresponding trajectories will allow for a more complete picture of the dynamic decision making process.

A particular strength of the rapid reaching paradigm described above is the ease with which an experimenter can modify the task to examine a wide variety of psychological and sensorimotor research questions. One simple, yet quite powerful manipulation is to replace the cue display containing only potential targets (i.e., black circles with an equal likelihood of being cued) with more complex visual stimuli. We denote the ease of this possible manipulation in Figure 2 by using red and blue boxes as indicators of (or placeholders for) arbitrary stimuli. Our first use of this flexibility was in examining how target salience interacted with the physical distribution of targets in space (Wood et al., 2011). In this study we were able to show that salience dominated early target competition (i.e., the hand was biased to the higher contrast targets, regardless of their physical distribution) but that later, after additional processing time, the physical distribution of the targets dominated their salience (i.e., the hand was drawn toward the side of space with more targets). As a second example, we have recently used Arabic numerals (e.g., “2”) in place of the corresponding number of potential targets in the cue displays in order to show profound differences in magnitude processing between non-symbolic and symbolic number formats (see Chapman et al., 2014). As a final example, we have recently replaced the target circles with other arbitrary shapes (e.g., squares, stars, etc.) and had participants learn a reward association between shape and value (Chapman et al., submitted-a,b). Even though the physical distribution of targets is identical in this case (e.g., it is always one on the left vs. one on the right), we find strong deviations toward shapes associated with high reward. In principle, there is no restriction on what visual stimuli the cue display can contain, including complex shapes, rich color images and moving stimuli; if there is good reason to believe there will be a bias in processing a certain visual stimulus compared to another, the visual stimuli can be inserted into the cue display and reach trajectories will likely provide a sensitive cognitive read-out of this biased processing.

A second modification on the rapid reaching task that we have explored in previous work is shifting it from a forced choice to a free choice paradigm (see Target Display options in Figure 2ii,iii). That is, in addition to having cases in which a final target is cued for the participant on a given trial (as described in the General Procedure above), we have also implemented cases in which, under the same rapid response constraints, participants are allowed to freely choose which of two (or more) options they will reach toward (Chapman et al., submitted-b). In this work, we have shown that the bias generated by a learned reward contingency is more visible in a free choice task than it is in the forced choice task. By implementing a free choice task, one can extend their investigations to examine not just automatic biasing behavior (in the case of forced choice tasks) but also how biases can affect deliberate (albeit rapid) choices. As such, this modification makes this type of paradigm directly amenable to researchers investigating decision-making processes, and the various types of cognitive biases that are known to affect one's choice behavior.

A last example of some of the ways in which the rapid reach paradigm can be easily modified to the benefit of the experimenter is to alter the timing of the task. In doing so, one can begin to probe the precise timeline and dynamics of cue display processing. As shown in Figure 2i, one example of this could be to manipulate the amount of time participants have to process a given cue display by introducing a delay between the cue display onset and the movement go-signal. This particular manipulation would have the effect of increasing the availability of cue display information for additional cognitive processing. Another example of this could be to introduce a delay between movement onset and the onset of the target display (see Figure 2ii). This particular manipulation would instead have the effect of ultimately decreasing cue display processing time. In a recent study we compared reaching under prototypical conditions (i.e., target display comes on a movement onset) to conditions in which participants had an additional 500 ms to view the cue display prior to the beep signaling movement onset (Wood et al., 2011, as in Figure 2i). In this study we showed that whereas the physical luminance of targets introduces the largest bias under rapid processing times (i.e., no delay), the number of targets introduces the largest bias under prolonged processing times (i.e., an additional 500 ms). In ongoing investigations, we are exploiting pre- and post-movement delays to explore the effects of a wide range of stimulus processing times on rapid reach trajectory biases. For example, in our work with reward contingencies, we have shown, using a wide range of delays, that positive reward information affects reach trajectories earlier than negative reward information (Chapman et al., submitted-b). This experiment, and the task in general, can include some cases in which the target display does not actually appear until some point during the rapid reach movement itself (as in Figure 2ii). These more extreme cases allow us to explore both the limits and extent of processing time and online reach control, and, combined with a range of other delay parameters, allow us to behaviorally map out a fairly precise timeline of the influences of various cognitive biasing factors.

One final point regarding modifications of the General Procedure concerns the issue of what one should take as a measure of non-spatial averaging or “baseline” behavior. That is, when quantifying the spatial averaging effects elicited by multi-target cue displays, what experimental conditions (and corresponding trajectories) should be used for comparison? In our past work we have most often used single target cue displays as our baseline measure (i.e., displays presenting only a single unfilled circle that is then filled-in at movement onset). However, we have also explored alternatives such as having baseline displays consisting of a single filled target that remains in that state throughout the entire trial (Chapman et al., 2010a). These two cases produce identical reaching behavior and, not surprisingly, show significant differences from trajectories toward multi-target cue displays.

The issue of measuring baseline behavior, however, becomes more complicated when one considers more diverse stimuli. As an example, in the previously mentioned experiment exploring the processing differences in numerical format, for target displays containing numerals, it was not a trivial matter to develop a cue display that accurately mimicked a corresponding single target cue display. Indeed, as we reported, although a cue display presenting “1” to the left of fixation and “0” to the right of fixation conveys the appropriate single-target information, the symbol “0” is still a visual object that must be initially processed, and our trajectory analysis suggested that “0” was still treated, to some small degree, as a potential target (see Chapman et al., 2014). We raise the issue of appropriate baseline selection here only as an exemplary illustration that any additional visual item in the cue display has the potential, at least initially, to compete for action selection.

### General analysis information

#### Reach trajectory preprocessing and extraction

**Figure 3:**
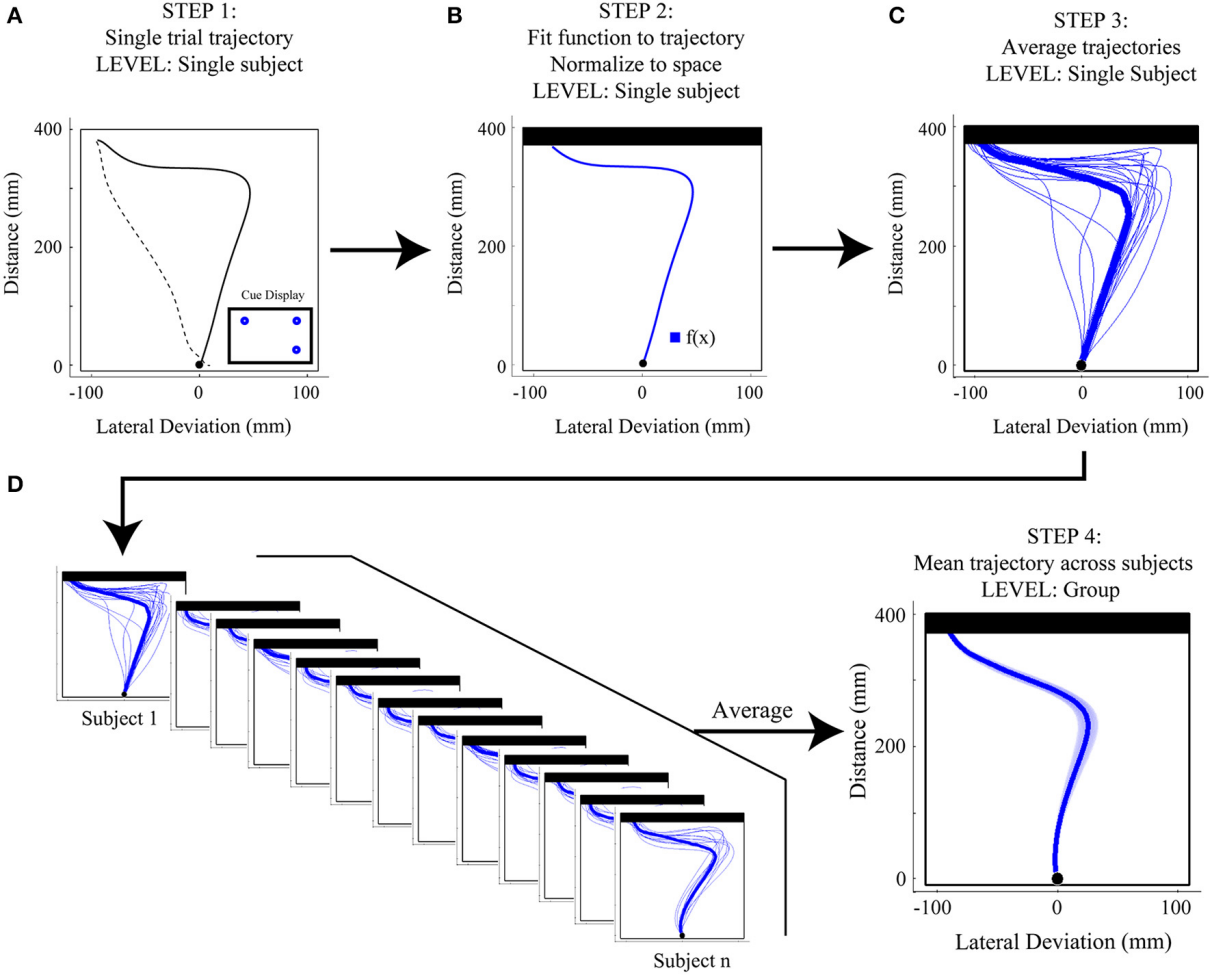
**Step-by-step overview of Functional Data Analysis (FDA) methods. (A)** Step 1: Single trial reach trajectories are extracted for a particular experimental condition [in the case here, a 1(high)v2 target display with the target on the left cued]. The full trajectory is denoted by a dashed black line and includes the hand returning to the start position, while the extracted reach is denoted by a thicker solid black line and includes only the movement of interest, from start position to touch screen. **(B)** Step 2: The single trial reach trajectory is then fit with a mathematical function [f(x)] and is then normalized to space (in the case here, with respect to touchscreen distance). The resulting space-normalized trajectory is denoted by the thicker blue trace. **(C)** Step 3: Single trial trajectories belonging to the same experimental condition are averaged, producing a single-subject average trajectory. Single trial trajectories are denoted by thin blue traces and the single-subject average trajectory is denoted by the thick blue trace. **(D)** Step 4: The single-subject average trajectories (at left) are then averaged across subjects to produce a mean group-level trajectory for the particular experimental condition (shown at right). At right, the shaded area around the darker trajectory represents the average standard error across subjects.

As depicted in Figure 3, the process of analyzing reach trajectory data starts at the level of an individual trial (Figures 3A,B), moves to the level of a participant average of many trials within a given experimental condition (Figure 3C), and finally to a group average of that condition, which represents the average of all participants' mean trajectories (Figure 3D). In order to analyze each individual trial it is first necessary to extract only the portion of that trial that corresponds to the movement of interest (in this case, the movement from the start button to the touch screen, see Figure 3A). To accomplish this, we first clean the data by filling in any small sections where a marker may have gone missing for a few frames. This is done as follows: First, if the second marker on the index finger is available and visible (i.e., not missing for those frames) we translate its data to the position of the missing marker to fill the missing frames. Second, if the second marker is not available, we employ either simple linear interpolation of the missing segment, or use the inpaint_nans function (available online at: http://www.mathworks.com/matlabcentral/fileexchange/4551) in Matlab, which provides a more sophisticated interpolation and better preserves the dynamic information of the reach. The full trajectory, with no missing data, is then filtered with a low-pass Butterworth filter (dual pass, 8–12 Hz cutoff, 2nd order) to remove any high frequency noise. We then translate all reaches such that the first reading of the trajectory is taken as the origin, and, if we have collected data from an external reference frame (e.g., from multiple makers on the touch screen), we rotate the data such that the straight ahead direction is the same for each trial (e.g., orthogonal to the plane of the touch screen). This translation and rotation compensates for any differences in reference frames arising due to errors in setup or recording and helps standardize across trials and participants. Following these steps, we can now extract the actual reach data and do so by first finding the point corresponding to the onset of movement. Here, we use the three-dimensional (i.e., vector) velocity (generated from the position data, and usually also low-pass filtered at 10–12 hz) and set the onset frame to be the first of four consecutive vector velocity readings of greater than 20 mm/s in which there was a total acceleration of 20 mm/s^2^ across the four points. We set the offset frame to be whichever of two events occurs first: the frame containing the maximum position value in the direction of the reach (i.e., the max reach extent) or the first frame in which the velocity drops below 20 mm/s.

#### Reach trajectory normalization

Once we have extracted clean, filtered and standardized data from a single trial, it is imperative to normalize the data such that we can generate averages across trials and, ultimately, participants. That is, since all trials will likely be defined by a different number of frames, they must be standardized so that they can be averaged together.

This issue of normalization is not as straightforward as it might seem. In many kinematic studies, trajectories are simply normalized to MT. That is, regardless of how long it took for the trajectory to be produced, it is resampled to have some common number of data points evenly spaced across its duration. However, in any study in which the experimental manipulation itself might introduce differences in MT, time-normalization can introduce artifacts into the data, resulting in either phantom significant effects or reduced real effects. This has been nicely demonstrated in a recent paper on grip aperture trajectory normalization (Whitwell and Goodale, 2013), and we have found that similar problems are encountered in reach trajectory data if it is time-normalized. As a general rule, data should only be normalized to a dimension that does not vary significantly across one's experimental conditions. For our purposes, this choice is simple: Since participants always start and end their reach movements at the same points in space (i.e., start button to touchscreen), we are safe to normalize across reach distance.

To allow for space normalization we use a set of tools known as FDA (Ramsay and Silverman, 2005). Put simply, every extracted spatial trajectory is fit with a mathematical function (see Figure 3B). Once fit, the mathematically represented trajectory is thereby scale invariant, and we can extract data from whatever points in space (or time) are desired. More technically, for each trial, the discrete data in the extracted reach trajectory is fit using B-splines. Spline functions are commonly used to fit motion data that are not strictly periodic (Ramsay et al., 1996; Ramsay, 2000; For an example of recent papers using a similar technique see Loehr and Palmer, 2007, 2009). In our work, order six splines are fit to each of the three dimensions (x, y, z) of the motion data with a spline at every data point. The data are then smoothed using a roughness penalty on the fourth derivative (λ = 10^−18^, within 0.00001 of the generalized cross-validation estimate, Ramsay and Silverman, 2005), which allows for control of the smoothness of the second derivative. This process generates the mathematical definition of each dimension of data (x, y, or z) across time.

To normalize to reach distance, we evaluate the reach direction (y) component of the reach at thousands (usually 2000) of equally spaced points in time. This ability to significantly oversample in time, afforded by the mathematical fit, is the critical step that allows one to move from time normalization to space normalization. To do this, we extract from this high resolution reach-distance trajectory the location and times that correspond to points that are equally spaced along the reach distance. Finally, we then extract the corresponding three dimensional data at these reach-distance-normalized time values to generate our final space-normalized trajectory. We usually extract between 100 and 200 reach-distance-normalized times, and as such, all of our trajectories will contain this same number of data points, and are thus easily averaged. In Figure 3C we show an example of a participant average for a given experimental condition generated from multiple normalized trials (one of which is shown in Figure 3B) and in Figure 3D we illustrate that multiple single participant mean trajectories are combined to produce a group average trajectory for a single experimental condition.

#### Trial screening

Once extracted and normalized, data are first screened to remove any trials with obvious recording errors. These errors include trials with too few data points (<100 ms of data), too much missing data (more than 100 ms of missing data) or a maximum reach distance that falls too short of the actual target distance (>5 cm). In our previous work, recording errors have generally led to the removal of 0–5% of all trials.

As described above, we apply strict criteria to ensure only fast and accurate reach trials are included for analysis. To make certain that the timing demands for our RT criteria are met, we remove trials that resulted in Too Early or Time Out errors (see section General Procedure Information). In our previous work, these have typically accounted for 0–5 and 5–10% of trial removal, respectively. To ensure that the demands for spatial pointing accuracy are met, we also remove trials that result in a Miss error, usually accounting for 5–15% of trial removal. (Of note, Miss errors increase as the spatial spread between potential targets increase. In paradigms with only two targets spaced ~20 cm apart, Miss errors usually result in <5% of trials being removed). Since participants can struggle with the rapid MT requirements of the task (i.e., often close to 50% of trials result in a Too Slow error) we are more lenient in removing these trials. Previously we have either removed the slowest 5% of trials, or, have removed trials that are excessively long (e.g., >850 ms, or twice the allowed MT duration) and more than two standard deviations above each individual participant's MT mean. The resulting removal of these Too Slow trials usually results in 0–5% of trials being discarded, and notably means that we include in our analysis some trials in which participants received “Too Slow” feedback at the end of the trial.

In sum, our trial screening procedure usually results in ~30% of trials being removed from analysis. As mentioned in the Participant section above, following trial screening we eliminate any participants with poor performance. Following trial and participant removal, we usually end up analyzing 75–85% of the trials completed by the included participants.

### Current study analysis information

#### Reach trajectory analysis

Trajectories in the current experiment were extracted and normalized using procedures identical to those described above, with the following parameters: position data was low-pass filtered with a 10 Hz cutoff and normalized reaches consisted of 200 data points.

#### Trial screening

In the current experiment, we removed 4.1% of trials due to recording error, 0.9% of trials for being Too Early, 8.7% of trials for resulting in a Time Out, 12.2% of trials that resulted in a Miss, and 3.0% of the trials that were executed the most slowly. In total, this meant that 28.9% of trials were removed from analysis. Following the removal of six participants for poor performance, we analyzed 76.9% of the remaining participants' data.

### General statistical comparisons

A challenge when working with reach trajectories is to develop sensitive analysis techniques that can allow for the detection of differences in trajectories throughout the course of the entire movement. Clearly, this is a challenge worth meeting because conventional single value representations of data, while often useful, can run the risk of over simplifying complex movements, or missing crucial areas of difference by restricting analysis to a single data point. Here we discuss the ways that we have used both functional comparisons and more conventional non-functional comparisons to test for statistical differences in reach trajectories.

#### Functional comparisons of trajectory data

**Figure 4:**
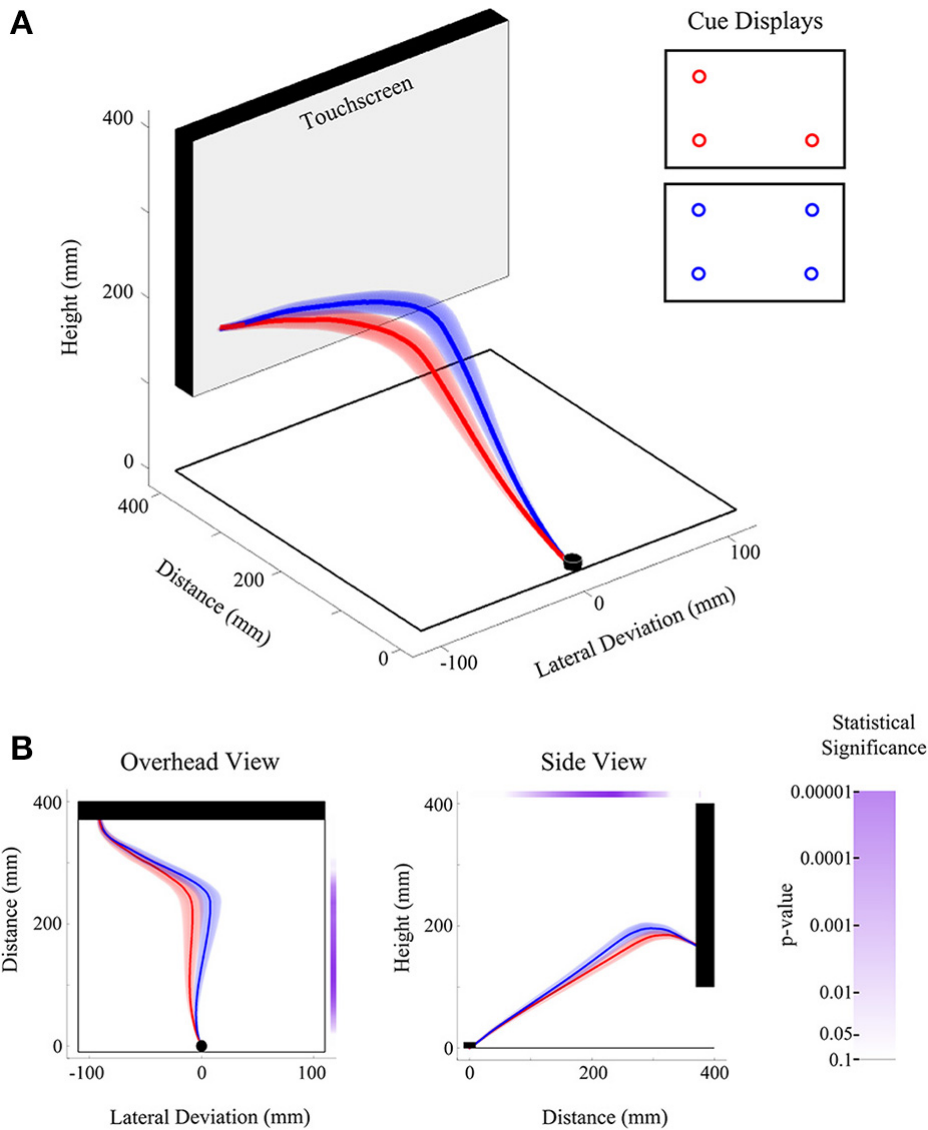
**Illustration of experiment results using FDA. (A)** Three-dimensional view of the group-averaged reach trajectories for 2v1(low) (red) and 2v2 (blue) target displays. Results are shown for trials on which only the bottom left target was selected as the final target. Shaded areas around the darker trajectories represent average standard errors across subjects. **(B)** Data is the same as in **(A)**, except shown from an overhead (left) and side (right) view. Significance bars (shown in purple at right and top for the overhead and side views, respectively) provide a measure of where there are statistical differences between red and blue trajectories in the lateral dimension (at left) and height dimension (at right). The color saturation of the significance bar denotes the statistical threshold. See the significance bar at far right for interpretation of statistical significance levels.

Working from the principles of FDA (Ramsay and Silverman, 2005), we have developed custom Matlab algorithms to run functional analysis of variance (fANOVA) statistics on space normalized reach trajectory data (for example data and analysis code, see Links at http://www.per.ualberta.ca/acelab/). A fANOVA is an extension of the traditional ANOVA (with only a single dependent variable across groups) that can be applied to continuous data (like the spline-fitted trajectories described here). Whereas a traditional ANOVA provides a single F-statistic that indicates differences among means, the fANOVA provides a functional F-statistic that shows not only if, but also where, and to what degree a set of functionally defined measures differ across conditions. It is important to recognize that the functional F-statistic (and indeed, each trajectory entered into the analysis) is a single data object—it is only meaningful when presented and discussed in its entirety (for an example, see Figure 4). Thus, it would be statistically inaccurate and devoid of theoretical meaning to employ a fANOVA to find where trajectories differ and then present an analysis over only that sample of data.

FANOVAs can be applied to any single dimension of data [e.g., lateral deviation (x) Figure 4B panel 1, or deviation in height (z) Figure 4B panel 2], though possible extensions of this analysis could include multivariate analysis, which considers more than one dimension at a time (e.g., a functional multivariate ANOVA). FANOVAs, like univariate ANOVAs, can be applied across multiple levels of an experimental factor, or used to investigate relationships between multiple factors and their interactions. In our own work we have exclusively employed repeated measures (RM) fANOVAs and have predominantly examined trajectory differences across two or more levels of a single experimental factor in the lateral deviation (x) dimension (the dimension that is typically manipulated in our experiments). It is worth noting that in the case of a fANOVA comparing only two trajectories (see Figure 4), the fANOVA is reduced to a functional-*t*-test, and is a useful way to investigate specific pairwise differences between experimental conditions.

#### Measuring bias—comparing reaches toward mirrored cue displays

**Figure 5:**
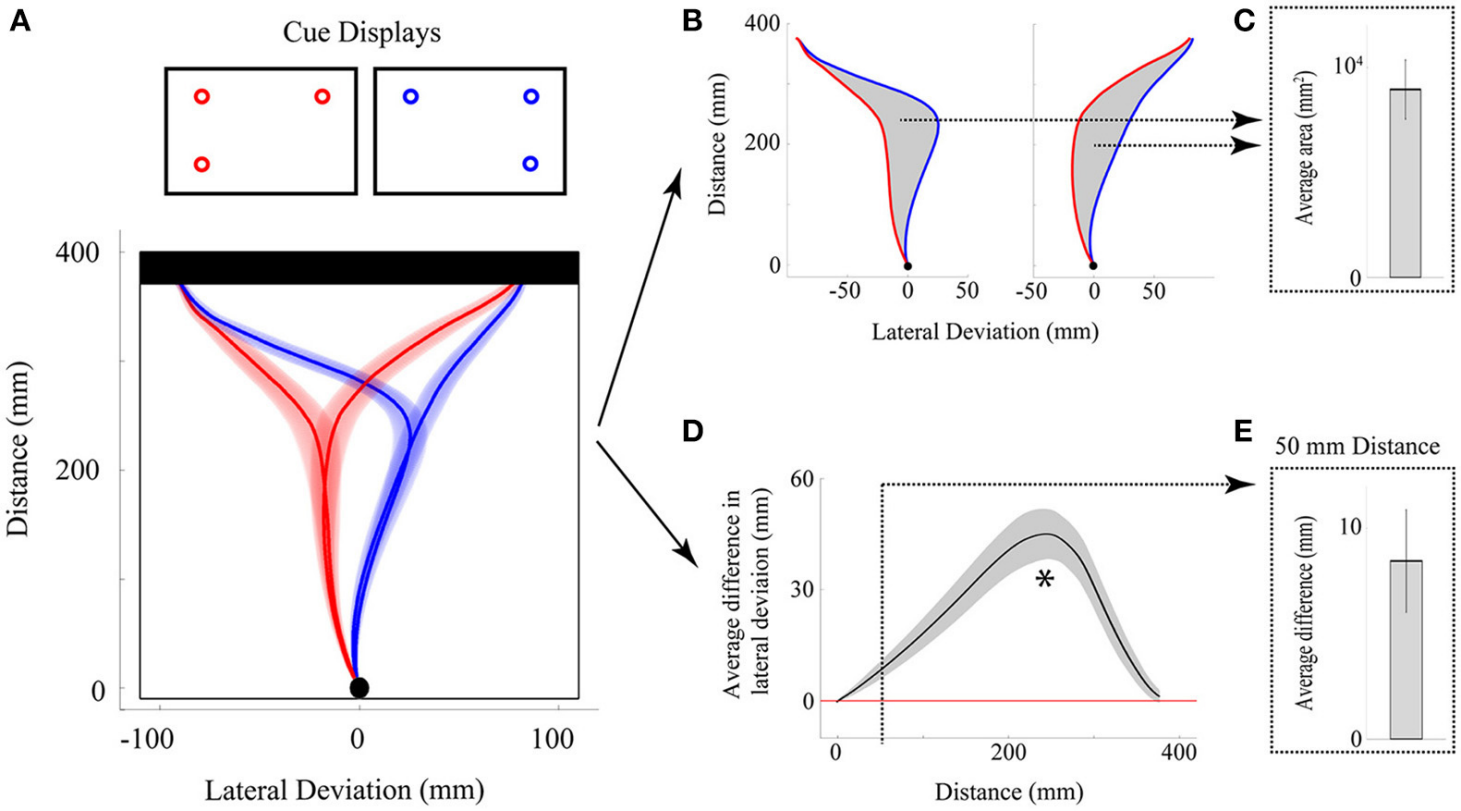
**Illustration of experiment results using non-functional measures. (A)** Overhead view of the group-averaged reach trajectories toward mirror displays [2v1(high) in red; 1(high)v2 in blue] for cases in which the top left or top right target positions were cued. Note that the direction of the initial movement trajectory is independent of which target position (top left or top right) is actually cued at movement onset. **(B,C)** Analysis of the average area between trajectory traces. The area between reach trajectories [gray shading in **(B)**] is computed for each reach endpoint position [top left or top right, **(B)**], averaged across endpoints and then individuals, and then plotted in bar graph form [in **(C)**]. Statistical significance is assessed by comparing the group-averaged area values to zero. Error bar shows the 95% confidence interval and, when above zero, denotes that the average areas between trajectories differed significantly (*p* < 0.05). **(D,E)** Analysis of the average lateral deviation between trajectory traces. The difference between reach trajectories over the entire distance of the movement is computed for each reach endpoint position and then averaged across endpoints and then individuals [black trace in **(D)**]. Gray shading around black trace in **(D)** shows 95% confidence intervals and, when above zero, denote that trajectories significantly (*p* < 0.05) diverged. Asterisk denotes the maximum lateral deviation difference observed (~60% of reach distance). The average lateral deviation difference at a time point of 50 mm distance is extracted and then plotted in bar graph form [in **(E)**]. Statistical significance is assessed by comparing the group-averaged deviation values to zero. Error bar in **(E)** shows the 95% confidence interval and since it is above zero, it denotes that the lateral deviation difference was significant (*p* < 0.05) even at this very early point in the reach.

While in principle any two conditions in an experiment can be compared (e.g., see Figure 4), we have developed one specific approach—an analysis of mirrored displays—that we believe is the most sensitive for testing the trajectory bias elicited by a specific cue display. The rationale for the analysis approach is simple: For a common target ratio (e.g., 2v1) we compare the effect of a trajectory bias toward the side of space containing the larger quantity (e.g., 2 in this example) when that quantity is on the left vs. right. To illustrate this idea, in Figure 5A we show reaches toward a 2v1 cue display (i.e., two potential targets on the left side of space vs. one target on the right side of space) and a 1v2 cue display (i.e., with the same target ratio but mirrored configuration). By comparing trajectories toward a common endpoint (left or right) for 2v1 trials against trajectories toward the same endpoint but for the mirrored 1v2 trials, one can fully capture and measure the extent to which a 66.6% probability of acting on one side of the display (i.e., 2:1 ratio) biases the initial trajectory in that direction. This general logic of mirror display comparisons can be extended to any cue display type (e.g., arbitrary shapes), assuming that it has a mirrored counterpart and is not exclusive to functional analysis (see below).

#### Non-functional comparisons of trajectory data

While we firmly believe that the best way to analyze and interpret reach trajectory data is to employ functional analyses, it can often be useful to simplify a given experimental result by presenting more familiar non-functional data. In our own work, when presenting the results of any non-functional measure we most commonly employ repeated measures ANOVAs (RM-ANOVAs) with the Greenhouse-Geisser (GG) correction for sphericity applied. When no *apriori* differences between conditions are hypothesized, we generally follow-up a significant RM-ANOVA with Bonferroni-corrected pairwise comparisons. Though there are a myriad of single-value metrics that can be used to represent deviations in reach trajectory (e.g., angular heading, absolute position, relative position, deviation velocity, etc.) here we discuss two measures that we have found to be particularly useful in summarizing trajectory findings.

***Area between reaches***. One measure that successfully tests if there is an aggregate difference between two trajectories is to compute the area between them (see Figures 5B,C). When used in conjunction with the mirror-display approach described above (see section Measuring Bias—Comparing Reaches Toward Mirrored Cue Displays), the area between trajectories can provide a highly sensitive single-value data summary of the maximum bias generated by a given cue display. For a given condition we can then find (for each individual) the average area difference generated on mirror-display trials with endpoints on the left *and* mirror-display trials with endpoints on the right. Importantly, this averaging allows us to collapse across both sides of space (i.e., collapse across reach endpoint) and removes any potential artifacts due to extraneous spatial biases (e.g., see section Right hand bias). Finally, we can then compare the mean of these average areas against zero using a *t*-test (or, graphically using 95% confidence intervals, see Figure 5C), allowing one to summarize, in a single plot, whether the bias generated by a given display is significant (95% CIs above zero) or not (95% CIs that cross zero).

Of course, it is worth recognizing that the area between *any* two curves is calculable, and we have recently begun using the area between trajectories when reaching to a right vs. left target as a measure of reach certainty. The logic of this comparison is as follows: In a condition in which a decision is certain (or, there is less competition between the two competing targets) the hand will move in a straight path toward the selected target, resulting in a large area between these leftward and rightward reaches. However, in a condition in which a decision is less certain (or, there is more competition between the two competing targets), the hand path will be more curved toward the selected target (the curved path resulting from competition with the opposite target). As a result, the area between leftward and rightward reaches in these less certain decision scenarios will be correspondingly smaller (Chapman et al., submitted-b).

***Lateral deviation differences***. Perhaps the most intuitive measure one can use to measure reach deviation is simply to look at the position of the hand at some point in time (or space) in order to infer where the reach was initially directed. When comparing two reaches, in order to provide an index of bias, this logic can be extended to examine the difference in reach position across two conditions. An illustration of this type of lateral deviation difference is depicted in Figures 5D,E. What is unclear from this measure, however, is exactly when in time (or space) it is most beneficial to extract this deviation information. Based on our previous work, if one is interested in extracting the maximum deviation difference generated by a given cue display, then this point is commonly found at ~60% of reach-distance (see asterisk in Figure 5D). However, since this point of maximum bias can be more than half way through a 400+ ms reach (i.e., ~200 ms), it means that enough time has elapsed for online control mechanisms to affect the trajectory. Indeed, previous behavioral work shows that in-flight trajectory corrections and adjustments can begin taking place around 100–150 ms (e.g., Soechting and Lacquaniti, 1983; Brenner and Smeets, 1997; Day and Lyon, 2000; Saijo et al., 2005; Franklin and Wolpert, 2008). Since in many of our tasks we are primarily interested in initial cue display processing, we have found it beneficial to examine deviation differences that occur before 100 ms. Based on our previous work, 100 ms in time generally corresponds to ~70 mm of reach distance. We therefore often find it best to compare lateral deviation differences somewhere in the first 50–100 mm of the reach distance in order to provide an estimate of the bias generated exclusively from cue display processing.

As with the area between curves, lateral deviation differences, when used in conjunction with the mirror display approach, can show great sensitivity to the biases generated by a given cue display, and can likewise be averaged across both end-left and end-right conditions to negate any specific effects due to side of space. Also, by extracting an average deviation difference for each individual and then comparing the average difference across individuals to zero, one can test whether a statistically significant bias was introduced by a cue-display at a particular point in time (or space). For an example of a difference extracted from mirror-displays at 50 mm, and its comparison to zero (via 95% CIs), see Figure 5E.

#### Reaction time

One of the advantages of reach tracking as an experimental tool is that, in addition to the rich information provided by continuous spatial trajectories, one can also attain more conventional measures of visual processing such as RT and MT (see below). Based on our task paradigm, we define RT as the time elapsed between the signal to move (auditory beep go-cue) and the first frame of detected motion (onset frame, as described above). When available, we have also sometimes defined RT as the time between the beep and button release (i.e., not motion defined). These two definitions of RT produce identical patterns of results, with the detected motion usually occurring earlier than the detection of a button release. Importantly, as described below, RT differences in our rapid reach tasks rarely provide insight into features of cognitive processing (i.e., they are almost always non-significant). As such, we have found it is not necessary to discuss RT findings when employing this task.

#### Movement time

We define MT as the time elapsed between the onset frame and offset frame (described above). We have sometimes also used the time elapsed between button release and a recorded touch screen hit. Again, these two definitions of MT produce identical patterns of results. Of course, RT and MT are not the only discrete temporal parameters that one can extract from a reach, and in some previous studies we have also examined different components of the reach velocity (e.g., peak velocity, time to peak velocity, and the relative time spent accelerating vs. decelerating). However, as we will describe below, all of these discrete temporal parameters, including MT, only appear to simply track the total path distance of a reach. The velocity profiles of rapid reach movements are fairly stable across conditions, and thus the only thing that affects MT is the actual distance traveled by the hand. As such, we have found that it is not necessary to discuss MT findings when using this task since the results are mostly redundant and often more confusing to unpack than the much more intuitive and easily visualized reach trajectory results.

### Current study statistical comparisons

In the current experiment we present examples of both functional (Figure 4) and non-functional (Figure 5) analyses of our reach trajectory data. These results, as well as an analysis of RT and MT, will be described below.

## Results

### General results information

#### Results presentation

We have found some ways of visualizing our data to be particularly useful in conveying both the functional and non-functional differences in reach trajectories. For functional data, we have found that showing a representation of the reach that highlights the dimension of comparison (e.g., Figure 4B) is important and is often complementary to a depiction of the full three-dimensional trajectory (e.g., Figure 4A). To convey the results of our fANOVAs, we use significance bars, placed next to, or above plots of a single dimension (e.g., lateral deviation or height) that denote where, throughout the reach, the trajectories being compared are statistically different. Moreover, we use the saturation of the significance bar to denote the degree of the significance of the comparison. As seen in Figure 4B, we usually denote a range of significance values from *p* = 0.10 (completely white) to *p* = 0.00001 (completely saturated).

Another way of showing the results of a functional comparison between only two trajectories (i.e., a functional *t*-test) is to plot how the difference between them evolves over the duration of the trajectory (for example see Figure 5D). By adding the confidence intervals (e.g., 95% CIs) around this difference, we show the full result of the functional *t*-test: When the error bars include zero, the difference is not significant and when they do not include zero, the difference is significant. This, of course, also applies to non-functional data (as described above) and as can be seen in Figures 5C,E, adding 95% CIs to the plot of a group mean difference relative to zero makes the result of the related *t*-test immediately visible in the graph.

Finally, when showing any group average in a RMs design, it is important to consider what variability most accurately captures the variability tested by the analysis. For example, showing between-subjects variability is meaningless since this variability is exactly what is factored out in an RM design. Instead, it is important to show some measure of average within-subject's variability. There are many good techniques for conveying variability appropriate for conventional RM-ANOVAs, but for our reach trajectories we opt to show the average individual participant standard error around the trajectory. That is, for each participant and condition we calculate the standard error of that participants trajectory data at each normalized point. We then average these standard errors across participants in order to generate the confidence “tubes” seen in our trajectory plots (see Figures 1B, 3D, 4A,B, 5A). These error tubes closely parallel the statistical comparisons being made and, as such, they help convey information concerning overall differences between trajectories.

#### Right hand bias

While many results of a study are usually unique to that particular study, there is one effect that emerges in almost every experiment we have performed to date: An overall trajectory bias toward targets on the right hand side of space. This effect is manifest in a variety of ways:

1. Biasing effects (e.g., deviation differences, area between curves, significance of fANOVA) to right hand targets appear smaller than biasing effects to left hand targets.
2. MTs to right hand targets are faster than MTs to left hand targets.
3. In cases in which participant behavior suggests they are guessing (e.g., rapid free choice), they are much more likely to choose right targets over left targets.

Evidence for this general right hand bias is perhaps most obvious in a study we conducted investigating the effect that repeatedly cuing a target consecutively at one spatial position had on initial trajectory biases (Chapman et al., 2010b). In this study, we found that participants' trajectories were biased toward the right target after only a single repetition at its location and were saturated (i.e., showed no further incremental biasing) after four repetitions at its location. In contrast, we found that trajectories were only significantly biased to the left target after two repetitions at its location and were not even fully saturated even after five repetitions. We interpret all of these results as indicating that, with all else being equal, participants are more attracted to the right target positions than the left target positions. Indeed, even separate labs employing variants of the paradigm have revealed this same general tendency in their participants (Stewart et al., 2013).

There are several potential reasons why a right hand bias would emerge in our studies. First, as we mentioned in the Participant section, we have tested only right-handed individuals using their right hand. Thus, for purely biomechanical reasons, it is likely easier for the right limb to reach toward a right target than a left target. More specifically, there are less biomechanical constraints to performing an online correction to the right target (requiring only an elbow extension) than left target (requiring an elbow flexion and a shoulder or trunk movement). Second, some neuroscientists theorize hemispheric specialization in the processing of targets and other visuomotor features of movement (regardless of, though potentially related to, handedness, for review see Serrien et al., 2006), and it could be the case that reaching actions are planned more effectively toward rightward than leftward targets. Whatever the case, this right hand bias has not been relevant to any of the empirical questions we have asked using this paradigm, and, as such, we have developed measures to mitigate its effects on our data (like collapsing across target endpoints for analysis, see above).

### Current study results information

#### Reach trajectories

The purpose of presenting data from the current experiment is more to serve as an example of the paradigm and analyses described, rather than to provide an exhaustive account of the biasing induced by targets distributed across two distinct spatial dimensions (i.e., x and z). Accordingly, we have elected to show data from two particular reach trajectory findings that, we believe, exemplify the strength of the paradigm and analysis. In addition, we also take this opportunity to briefly discuss how these findings add to our empirical knowledge.

The first of these findings, depicted in Figure 4, shows the trajectory deviation differences between a three-target cue display (red trace Figure 4, 2 targets left, 1 target bottom right) and the four target display (blue trace, Figure 4), both ending at the bottom left target position. As predicted from a model of multiple motor plan competition (Cisek, 2006, 2007 see also Discussion) and fully consistent with the probabilistic spatial distribution of potential targets, we show that with the addition of the fourth potential target to the cue display, the hand path subtly shifts toward that fourth target in both the lateral (first panel Figure 4B) and height dimensions (second panel Figure 4B). Following from our discussion of General analysis and results presentation, we denote the results of a fANOVA comparing these two trajectories (red vs. blue traces) using a significance bar (see purple bars in Figure 4B). As can be clearly seen, the difference in trajectories in both the x and z dimensions arising due to the probability of acting at the fourth target location emerges early, is strongest through the middle part of the trajectory and, of course, is at or near zero at the end of the reach (since both trajectories converge upon a common endpoint). This pattern of results is fully consistent with our previous work and is representative of the trajectory effects seen across the entirety of the current study. That is, the initial hand trajectory always follows a path which averages between the spatial locations of the potential targets in the cue-display, up to a limit of about four potential targets (see Gallivan et al., 2011).

The second example trajectory analysis is depicted in Figure 5 and further demonstrates the substantial effect that the number of potential targets has on initial reach trajectory. Here we show reaches toward 2v1(high) and 1(high)v2 displays that end both on the left and right (Figure 4A; i.e., mirror-image displays). To quantify the deviation differences toward these displays we show the statistics associated with two complementary analysis approaches: (1) the area between curves (Figures 5B,C) and, (2) the lateral deviation differences at 50 mm (Figures 5D,E). Notably, both of these measures allow us to collapse across leftward and rightward endpoint reaches to derive a group average of the initial bias toward this cue display that significantly deviates from zero (see 95% CIs in the bar plots in Figures 5C,E). Again, both these measures convey the same result as our previous work: The hand is attracted toward the side of space with more potential targets.

#### Reaction time

To test the extent to which RT measures might reveal an effect of multiple targets in this paradigm (in accordance with Hick's law, 1952, whereby simple choice RTs scale with the number of available choices), we conducted a one way RM-ANOVA with number of targets as a factor (1, 2, 3 or 4). Notably, this test was not significant [*F*_(2.74, 63.11)_ = 2.23, *p* = 0.098], and all RTs were clustered around the average value of 242.5 ms. This finding replicates the general insensitivity of RT measures previously reported using our task (Chapman et al., 2010a,b, 2014; Gallivan et al., 2011; Wood et al., 2011; Milne et al., 2013; Stewart et al., 2013), though some of our previous work has revealed a basic RT advantage for single (baseline) vs. multiple (experimental) target trials. To more thoroughly explore any RT effects in the current data, we also further conducted a one way RM-ANOVA across the thirteen possible cue-displays. Here we did find a significant RT effect [*F*_(7.43, 170.99)_ = 4.00, *p* < 0.001]. To follow this up, we ran separate RM-ANOVAs on the single target cue displays (four total) and the multiple target cue-displays (nine total). Here, only the single target cue-displays showed significant differences in RT [*F*_(2.75, 63.26)_ = 5.88, *p* < 0.005] while there was no effect on RT across the multiple target displays [*F*_(5.63, 129.59)_ = 1.60, *p* = 0.16]. Pairwise comparisons of the single target trials revealed that average RTs were faster for a target appearing in the top right location (238.7 ms) than a target appearing at the top left location (249.6 ms) or bottom left location (248.8 ms). Targets in the bottom right location resulted in intermediate RTs (243.8 ms) and were not significantly different than any other location. This finding indicates that the right hand bias, discussed above, extends to RTs for single targets. Importantly, however, RTs for trials involving more than one target were not statistically different, demonstrating that RT itself provides a poor measure of the competition between targets in this rapid reach paradigm, and thereby validating our usual exclusion of discussions concerning RT data.

#### Movement time

To explore any MT effects in the trajectory data, we ran a 2-factor, (4 × 4, Number of targets × End location) RM-ANOVA. This revealed significant main effects of Number of targets [*F*_(1.39, 32.07)_ = 368.96, *p* < 0.001] and End location [*F*_(1.89, 43.38)_ = 35.68, *p* < 0.001], as well as a significant interaction between the two [*F*_(4.96, 113.99)_ = 2.86, *p* < 0.05]. To follow this up, we ran separate RM-ANOVAs on the single target (baseline) trials (one-way ANOVA across the four end locations) and the multiple target trials (two-factor RM-ANOVA, 3 × 4, Number of targets × Endpoint).

MT analysis of the single target trials revealed a significant effect of end location [*F*_(2.57, 59.10)_ = 34.41, *p* < 0.001]. All pairwise follow-up comparisons were significant and showed that reaches toward the bottom right location were executed the fastest (340.4 ms), followed by reaches toward the top right location (348.3 ms), then reaches to the bottom left (355.4 ms) and finally the slowest reaches were toward the top left location (365.8 ms). This finding again demonstrates that right-handed participants, in general, show an advantage for acting on targets on the right (indicative of the right hand bias discussed above). Also, the reduced MTs for bottom position targets are indicative of the fact that the bottom position targets are located physically closer to the starting position of the hand.

The RM-ANOVA comparing MTs across only multi-target trials revealed a significant main effect of number of targets [*F*_(1.58, 36.22)_ = 148.59, *p* < 0.001] and end location [*F*_(1.78, 40.94)_ = 26.74, *p* < 0.001] but no significant interaction [*F*_(4.24, 97.60)_ = 1.49, *p* = 0.21]. All pairwise comparisons across the number of targets were significant and revealed that reaches were executed more quickly on trials with two targets (421.3 ms) than trials with three targets (447.9 ms), which in turn were faster than trials with four targets (457.6 ms). This finding is evident in the trajectories themselves, which show that, on average, the amount of physical distance required to correct a movement from its initial trajectory heading to the final target position is the largest with four targets, less with three targets, and the least with two targets. Pairwise comparisons across reach endpoint replicated the single target results noted above by showing that reaches were fastest to targets on the bottom right (426.0 ms) and top right (429.2 ms) than to targets on the bottom left (450.2 ms), with the slowest reaches to top left targets (463.8 ms). Again, this pattern of MT effects is fully consistent with a right hand bias and the shorter path length required to reach the bottom target locations. The finding that reaches toward multi-target displays take significantly longer than reaches toward single target displays is not surprising. This reflects the fact that multi-target displays, in which the initial trajectory follows a path that spatially averages between targets, requires an online correction to the cued target location and thus, a much longer physical distance to be traveled than the direct, straighter path allowed for by the single target trials.

In sum, the current experiment replicates our previous observations that MT measures simply track the physical distance to be covered in the trajectory, in particular, and primarily provide information redundant to that resulting from a direct analysis of the spatial reach trajectories themselves, in general. Moreover, it seems clear that MT effects can often be more complicated to understand and less sensitive to the features of cognitive processing than the corresponding spatial trajectory effects.

## Discussion

In this paper we detail a rapid reach paradigm developed to provide observable evidence of competing movement planning processes known to occur at the neural level (for reviews, see Cisek, 2007, 2012; Cisek and Kalaska, 2010). This paradigm builds and expands upon a recent surge of related behavioral work demonstrating the power of continuous movements to reveal evolving inner cognitive states (for reviews, see Song and Nakayama, 2009; Freeman et al., 2011). We believe that the effectiveness of the rapid reaching task relies on two key components: First, that participants both react and move very quickly, and second, that participants initiate their movement prior to knowing which of several potential targets will be selected as the final target for action. Equally, the sensitivity of the task relies on our ability to extract meaningful differences between reach trajectories that capture their full complexity. As described, we have a developed a set of tools relying on FDA (Ramsay and Silverman, 2005) as well as complementary non-functional statistics that allow for summarizing (and visualizing) the observed results. To demonstrate the efficacy and sensitivity of the task and analyses, we describe the results from a new experiment showing that initial hand paths are biased both horizontally and vertically toward potential targets that are spaced equally along both dimensions.

**Figure 6:**
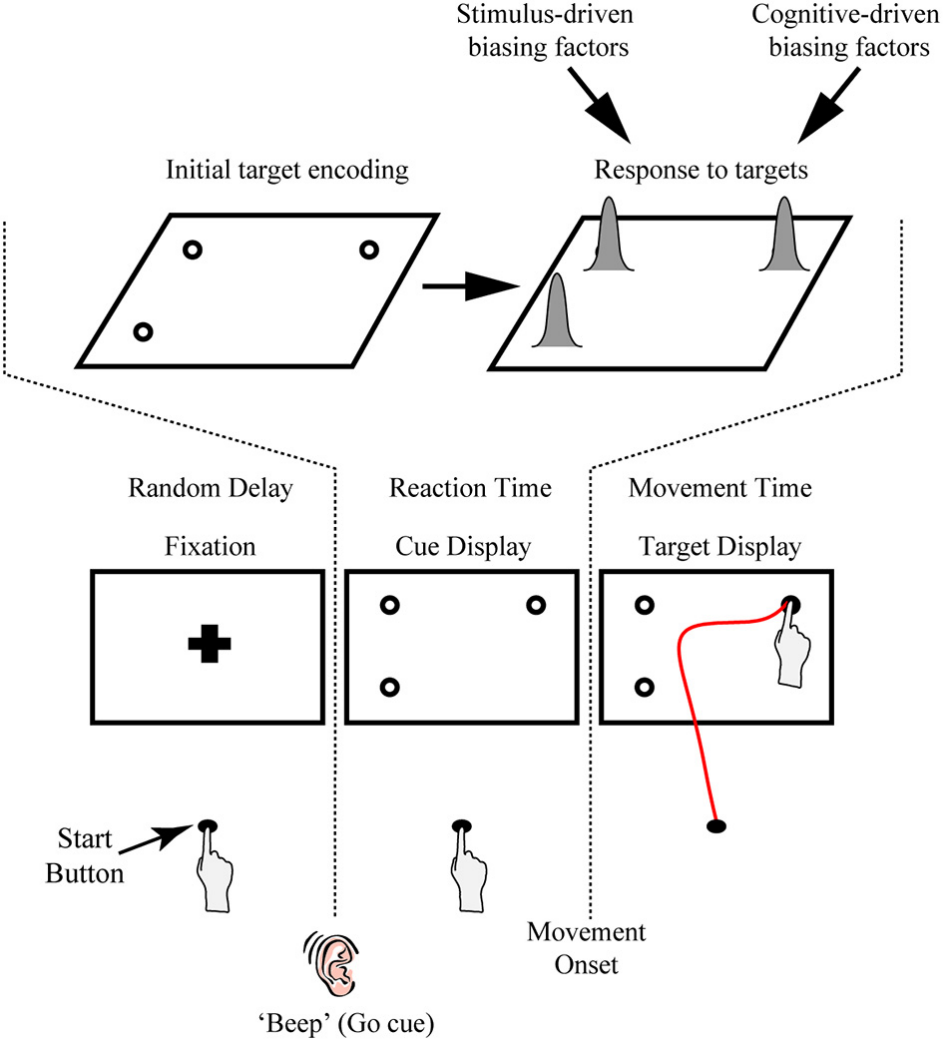
**Proposed descriptive model explaining initial reach trajectory biases. Bottom:** General timing of the rapid reach task, illustrated from a view behind the participant's hand. Red trace in the right panel denotes an exemplar trajectory toward the target display on trials in which the right target was cued. **Top:** Proposed temporal evolution of neural responses to the cue display prior to movement. Gaussian distributions (in gray) represent “hills” of activity that bias early reach trajectory responses. These “attentional landscapes” and associated “relevance maps” likely have a myriad of inputs, including stimulus-driven and cognitively-driven biasing factors, which can be assessed through the careful analysis of reach trajectories described here.

As the development of our task was originally aimed at providing behavioral evidence for the specification of multiple competing movement plans (Chapman et al., 2010a), it should come as no surprise that we situate our results within theoretical frameworks describing how parallel movement planning processes are implemented in the brain and how they evolve through behavior. Specifically, our findings support frameworks positing that (1) multiple relevant objects in the environment are initially processed as targets of potential action and, (2) that multiple potential actions plans to those targets compete for overt execution over the course of a movement (Cisek, 2007; Cisek and Kalaska, 2010). This competition has been suggested to result in a constantly shifting attentional landscape in which “hills” represent behaviorally relevant objects and “valleys” represent objects to be avoided (Baldauf and Deubel, 2010). Since the hand is the physical effector that actually traverses these complex landscapes (and is attracted toward hills and avoids valleys), it stands to reason that detailed measurements of its path through three-dimensional space can provide a sensitive read-out of the current state of the landscape. Figure 6 provides a schematic illustration of how this kind of framework might be used to account for the empirical data presented here. In this framework each potential target gives rise to a “hill” of activity that competes for selection. Within the context of the current experiment, these hills would be arranged along both the vertical and horizontal dimension, and the rapidly executed initial hand movement averages along both dimensions to produce the types of trajectories we report here.

Several researchers contend that these “attentional landscapes” are manifest in the brain as a relevance, or priority map (Colby and Goldberg, 1999; Kusunoki et al., 2000; Itti and Koch, 2001; Fecteau and Munoz, 2006; Gottlieb, 2007; Bisley and Goldberg, 2010), in which the strength of neural activity at each target location reflects the likelihood, or bias, of acting upon that object (or, in some models, one's allocation of attention to that object). In everyday life, the decision to act on a specific object likely occurs from the resolution of the competition between the different mapped object locations (Cisek, 2006, 2007). As such, most movements performed on a daily basis are directed straight toward a single, selected location. However, as described above, a key feature of our task is that we force movements to be initiated before the competition between targets has be resolved (i.e., before the final target has been cued). In doing so, we are able to reveal, through the early component of the reach trajectory, the ongoing competition between mapped locations and through this, obtain a direct read-out of the evolving state of the priority map.

### Hand, eye, and mouse trajectories

Before discussing some specific ways in which one can extend the rapid reaching task, it can be informative to consider how other types of action-related responses may differ from reaching. As noted at the outset in the Introduction, both eye movements (see Van Der Stigchel et al., 2006 for review) and mouse cursor trajectories (see Freeman et al., 2011 for review) have been frequently used to explore the dynamics of decision making processes. We believe that these continuous measures have and will continue to provide important insights into the representation of competing action options and we encourage their use. Acting directly on targets with the hand, however, is different in important ways from both eye and mouse cursor movements. With respect to mouse cursor movements, while it is the case that use of a computer mouse is natural for many of us, it necessitates complicated visuomotor transformations to map mouse movements in the horizontal plane to observed cursor movements in the vertical plane. In addition, displacements of the computer mouse are not mapped to visual space in a one-to-one fashion. Often it is the case, depending on one's computer settings, that small amplitude mouse movement are transformed into large amplitude cursor movements, and likewise, low velocity mouse movements are often transformed into high velocity cursor movements. Furthermore, cursor movements are, by definition, restricted to a two dimensional plane; this of course is far removed from the dynamic three dimensional world that we actually move in, and that the reaches (as in our task) are performed in. In fact, the full three dimensional spatial competition observed in the current paper would have been difficult, if not impossible, to observe with mouse trajectories. This is not to say, however, that mouse trajectories may not ultimately reveal the same competitive biases as observed in reach behavior, but it does raise important empirical questions about the difference between mouse and reach movements and the ecological validity of these two behaviors for studying brain processes that have been evolutionarily shaped to deal with the planning and execution of actions in a real three dimensional world.

With respect to eye movements, we recognize that a detailed discussion regarding the difference between eye and hand trajectories is beyond the scope of the current Methods article. Following from above, it is worth noting, however, one fundamental difference between the two effectors: individuals use their hands to physically interact with and act on objects in the world whereas they use their eyes to gather information about objects/stimuli in the world. In an important recent review (Gottlieb, 2012), it is argued that the planning of eye movements is largely driven by “attention for learning.” That is, we fixate targets that are likely to give us information about the task at hand. By comparison, the planning of hand movements is largely driven by “attention for action.” That is, we tend to act on objects (or act in a way toward objects) in cases in which our ability to predict the outcome is most certain; this often means we tend to move in a way and toward objects that have previously produced a good outcome. Many times, these two different attentional biases will direct the eye and hand to the same object. For example, we might move our eyes toward a glass of wine to ensure it is the correct object, and then move our hand toward that glass because we enjoy the taste of wine (also, and again beyond the scope of this discussion, the eye can be used to provide feedback about the hand movement itself). However, there are also times when the goals of the eye and hand may differ; for example, we might want to look at the traffic light to see if it is switching from green to yellow, but we neither reach toward the traffic light, nor do we always perform the same motoric action once the light color is determined (e.g., on a green light I might do nothing, but on a yellow light I might down-shift the car and slow down). This brief discussion merely highlights some of the important differences that exist between hand, eye, and mouse cursor movements, and serves to motivate future work that considers all response domains in order to provide a comprehensive understanding of human decision making and action.

### Extensions of the task and future work

Despite being relatively new, the potential for this task to be exploited in other lines of research is already being realized. As an example, recent work employing a variant of the task has shown that the spatial averaging effects observed in reach trajectories also extends to the orientation of potential targets. Stewart et al. (2013) employed a task in which participants moved a hand-held rectangular tool toward multiple competing rectangular targets of varying location and orientation prior to one of those targets being selected after movement onset. Interestingly, the researchers found that both the initial hand direction and orientation were biased, respectively, by potential target locations and orientations, suggesting that fully elaborated target-directed movements, not just their directions, are specified in cases of target uncertainty.

In our own work, we are beginning to explore how the task can be used to examine a variety of stimulus- and cognitively-driven factors believed to effect motor plan competition, specifically, and relevance map encoding, more generally. As depicted in Figure 6, we envision that relevance maps can be biased by a variety of inputs, each of which can independently modulate the height (and therefore, behavioral relevance) of target activations in the map. As an example, in a rapid competition between a dim, poorly discriminated target and a bright, easily discriminated target, it seems obvious that the bright target should be represented earlier in the location map and more robustly (see, for example, Wood et al., 2011). This represents an example of a stimulus-driven bias. As another example, in an experiment in which one object is arbitrarily associated with a high probability of reward and another is associated with no, or a low probability of reward, we might similarly predict that the positively rewarding object be represented earlier and more robustly in the location map. This would be an example of a cognitively driven bias. Evidence from non-movement tasks (for reviews, see Anderson et al., 2013; Chelazzi et al., 2013) and our own recent results using reach behavior provide support for the notion that arbitrary learned reward associations do indeed act as a cognitive bias of stimulus processing. Of course, these two types of biasing factors (i.e., stimulus- vs. cognitively driven) are not mutually exclusive, nor are they by any means exhaustive. One could imagine that through learning, a “high-level” cognitive bias like an association between reward and color could come to affect the way in which color is initially processed at the “low-level” of stimulus responses. Likewise, there are any number of other routes and pathways through which biases can be introduced into the relevance maps, including through one's emotional responses, individual preferences and categorizations, retrieval of memories, etc. to name but a few. Given the flexibility of this general task, we believe it will be possible to insert nearly any category of stimuli (e.g., rewarding stimuli, pictures that are more or less personally appealing, well remembered or poorly encoded items, etc.) into the cue display and examine the trajectory biases they elicit, and by inference, how they influence relevance map encoding.

Notably, the rapid reach task offers the potential to read-out not just the current state of a relevance map, but also how the map is formed and shaped across time. As mentioned in the Extensions to Procedure section (section Extensions of Procedure), the rapid reach task, with only minor modifications, can be used to chart a detailed timeline of target encoding. For example, we previously referred to a result in which we showed that rapid reach responses were biased heavily by stimulus intensity, but after a 500 ms delay, these same responses were biased only by stimulus probability (see Wood et al., 2011). This finding indicates that somewhere within this 500 ms time window the “low-level” stimulus intensity bias was gradually replaced by the “higher-level” stimulus probability bias. It would be a relatively simple and intuitive extension of the current paradigm to vary this temporal delay to see how this transition occurs across these 500 ms, and also, how it might differ between individuals.

This specific example illustrates two important points: First, by varying the viewing time of the cue display one can examine the specific time course by which *any* factor biases neural competition and the subsequent resolution, or decision, between options. Second, sensitive reach metrics can serve as an important vehicle for exploring individual differences. In a recent study, we in fact combined both these two points and used a rapid reaching task to reveal individual differences in participants' weighting of two different biasing factors (Chapman et al., submitted-a). Specifically, we examined to what degree participants were biased toward shapes associated with high value compared to shapes associated with a high probability of selection. Even though at a group level these biasing factors appeared to contribute approximately equally in attracting the hand toward a target, we found that each individual had a relative weighting of these two factors that was almost entirely unique. Thus, this approach provided more insight into understanding their specific decision making biases than by simply analyzing the group statistics.

Another important avenue for future work involving this task is in computational modeling. Since hand movement paths provide an observable index of the ongoing competition between potential targets, results from reaching tasks readily provide a rich data set for modeling work. This has recently been elegantly demonstrated by Resulaj et al. (2009) who used reach trajectories to develop a computational model accounting for the timing and accuracy of initial decisions and why, on a subset of trials, participants altered their initial decisions. In this task, participants were required to make decisions on the direction of motion for a centrally located random dot motion array and indicate this decision by moving the handle of a robotic manipulandum to either a leftward or rightward target. They found that participants can exploit stimulus information in the sensory processing pipeline to subsequently reverse or reaffirm their initial decision and included a drift-diffusion model to provide a mechanistic account for this “change of mind” behavior. Other work has extended the framework of optimal control (Todorov and Jordan, 2002; for review, see Scott, 2004) to model reaching behavior in tasks like ours and show that goal competition (and the initial trajectory vectors) reflects a natural by-product of dealing with goal uncertainty (Christopoulos and Schrater, 2013) and provide a mechanistic account of intelligent rapid decision-making processes that can occur after movement initiation (Nashed et al., 2014). Recognizing that encoding in the relevance map should reflect the weighting factors from different input channels, we predict that, in addition to the modeling examples given above, it should also be possible to use rapid reach behavior to model what the responses may look like on each of these channels. As an example, one can model what types of stimulus response (i.e., the responses that *result* in the map activity) differences must exist between a target that generates strong biases toward its location vs. another that only weakly attracts the hand, or alternatively, actually results in biases away from its location (Chapman et al., submitted-b).

Finally, one further avenue that we believe is worth exploring concerns the degree to which our spatial averaging effects rely on the rapid responses that we have thus far enforced (i.e., constrained RT and MT criteria). That is, while the timing constraints of our task ensure that the resultant reach trajectory reflects the byproduct of an evolving (largely non-conscious) competition between potential targets, it is possible that even natural movements, without any time demands, might reveal some of the same information. As a simple example, imagine that you are pointing to a menu item at a restaurant to indicate your preference to the waiter. Even under these unconstrained timing conditions, recent pilot work from our group suggests that the trajectory of the hand (in this example, the finger) would reveal the confidence of your choice (Truong et al., 2013). For instance, if you are quite sure that you would like to order a steak, then your associated pointing movement will be rapid and direct. Conversely, if you are less certain about choosing the steak (e.g., you are also seriously considering the chicken), but nevertheless ultimately select the steak as your choice, your movement toward this menu item will be slower and less direct. The capacity to relax the timing constraints of the task, while still being able to measure the competition between choice options, will allow the paradigm to be even more generally applied.

## Conclusions

Here we have provided a detailed overview of our rapid reach paradigm and demonstrated its implementation in a new experiment. We have also used this opportunity to describe, in significant detail, some of the functional and non-functional analysis methods that we have found to be important in quantifying subtle trajectory deviation effects. Taken together, the current findings and that of our past work show that rapid reach movements, under cases of target uncertainty, have the capacity to provide a real-time read-out of an evolving neural competition between potential targets/stimuli and hold promise for investigating a wide range of cognitive states.

### Conflict of interest statement

The authors declare that the research was conducted in the absence of any commercial or financial relationships that could be construed as a potential conflict of interest.
